# Early Warning Systems for Acute Kidney Injury Prediction Before Clinical Onset: A Systematic Review of 24-48 Hour Risk Stratification Models

**DOI:** 10.7759/cureus.117931

**Published:** 2026-10-14

**Authors:** Manal Osman Alata Bakhit, Eiman Elzein Abdelrahman Elsheikh, Shaza Elsayed, Fatima Elgasim Elzein Elnagar, Husamedien Abas Gadalla Fdlalmolla, Mohamed Abdelgader Omer Farah, Mohammed Ateia Alawad Mohammed, Hanady ME M Osman, Safwa Obeid Eltreifi Obeid

**Affiliations:** 1 Nephrology, Prince Mohammed bin Abdulaziz Hospital, Madinah, SAU; 2 Acute Medicine, Ashford and St Peter’s Hospitals NHS Foundation Trust, Chertsey, GBR; 3 Rheumatology, Croydon Health Services NHS Trust, London, GBR; 4 General Medicine, University of Khartoum, Khartoum, SDN; 5 Nephrology, Noble's Hospital, Douglas, IMN; 6 Nephrology, Sligo General University Hospital, Sligo, IRL; 7 Nephrology, Jaber Al-Ahmad Hospital, Kuwait Ministry of Health, Hawalli, KWT; 8 Quality Improvement and Patient Safety, Najran Armed Forces Hospital, Ministry of Defense Health Services, Najran, SAU; 9 Nephrology, Mater Misericordiae University Hospital, Dublin, IRL

**Keywords:** acute kidney injury, clinical decision support, early warning system, machine learning, prediction model, probast

## Abstract

Acute kidney injury (AKI) is usually recognised only after serum creatinine has risen, when the chance to prevent it has largely passed. Electronic early warning models that predict AKI 24-48 hours before it meets diagnostic criteria could leave time to act. Following Preferred Reporting Items for Systematic Reviews and Meta-Analyses (PRISMA) 2020, we searched PubMed, Web of Science, Scopus, Embase, and Cumulative Index to Nursing and Allied Health Literature (CINAHL) for peer-reviewed primary studies published between January 2021 and September 2026. Eligible studies developed or validated models predicting incident AKI at a fixed 24- or 48-hour horizon in unselected hospitalised or intensive care patients and reported discrimination at that horizon. Data were extracted using the Checklist for Critical Appraisal and Data Extraction for Systematic Reviews of Prediction Modelling Studies (CHARMS) checklist, and risk of bias was assessed with the Prediction model Risk Of Bias ASsessment Tool (PROBAST).

Of 947 records, 13 studies were included. They came from the United States, China, Taiwan, Singapore, Sweden, and multinational consortia and together analysed about 623,000 admissions or encounters. Six studied intensive care patients, six studied general or hospital-wide inpatients (one of them in children), and one did not report its setting. Gradient-boosted trees and deep sequence models were the most common algorithms. Internal area under the receiver operating characteristic curve (AUROC) ranged from 0.72 to 0.99. External AUROCs ranged from 0.58 to 0.87 and were usually lower. Only two studies formally assessed calibration, only one included prospective validation, and none evaluated the effect of alerts on patient outcomes. Overall risk of bias was low in five studies, unclear in six, and high in two.

Models that predict AKI 24-48 hours ahead achieve moderate-to-good discrimination, but performance falls on external validation. Reporting of calibration and evidence of clinical impact remain scarce. Prospective, impact-focused evaluation should come before routine deployment.

## Introduction and background

Acute kidney injury (AKI) is an abrupt loss of kidney function that affects about one in five hospitalised adults worldwide and more than half of patients in intensive care units (ICUs) [1-4]. Even small rises in serum creatinine are independently associated with higher mortality, longer hospital stays and higher costs, and survivors remain at risk of chronic kidney disease [1,5]. Because the Kidney Disease: Improving Global Outcomes (KDIGO) definition relies on changes in serum creatinine and urine output, AKI is usually diagnosed after injury is established [2].

Serum creatinine is a lagging marker. It can take 24-48 hours to rise after a fall in glomerular filtration, which narrows the window for kidney-protective measures such as avoiding nephrotoxins, optimising haemodynamics and adjusting drug doses [6]. Electronic health records (EHRs) now capture vital signs, laboratory results and treatments continuously. This has driven the development of machine learning models that estimate AKI risk hours to days ahead [7,8]. A landmark deep learning model, for example, predicted 55.8% of all inpatient AKI episodes up to 48 hours in advance [8].

Detection alone does not improve outcomes. A multicentre randomised trial of creatinine-based AKI alerts showed no overall benefit and possible harm in some hospitals [9], which underlines the need for predictions that come early enough to change management. In this review, a prediction model is an algorithm that estimates AKI risk, whereas an early warning system also requires an alert threshold, a defined clinical response and evaluation of its effect on care; strong predictive performance alone therefore does not make a model an effective warning system. Since 2021, many AKI early warning models with 24-48-hour horizons have been published, but their performance, transportability and methodological quality have not been synthesised in one place. This systematic review therefore aimed to identify, describe and critically appraise original studies of models that stratify AKI risk 24-48 hours before clinical onset, which form the predictive component of such systems.

## Review

Materials and methods

Protocol and Reporting

This review followed the Preferred Reporting Items for Systematic Reviews and Meta-Analyses (PRISMA) 2020 statement [10]. The protocol was not prospectively registered because the review was planned and completed within a short timeframe; the eligibility criteria, extraction items, and risk-of-bias approach were nevertheless agreed upon before screening began.

Eligibility Criteria 

Eligibility was defined with the PICOS (Population, Intervention, Comparator, Outcomes, Study Design) framework (Table 1). Eligible studies developed or validated a multivariable model that predicted incident AKI at a fixed 24-hour or 48-hour horizon from the time of prediction, in unselected adult or paediatric inpatients or ICU patients. Studies also had to report discrimination (the area under the receiver operating characteristic curve (AUROC)) for that horizon. Only English-language studies published from January 1, 2021, onwards were eligible. This start date was chosen deliberately to appraise contemporary models developed with current EHR infrastructure and modern machine learning methods; influential earlier models [7,8] are therefore discussed as context only, and the review is not intended to cover all AKI prediction models.

**Table 1:** Eligibility criteria (PICOS framework) AKI: acute kidney injury; AUROC: area under the receiver operating characteristic curve; ICU: intensive care unit; KDIGO: Kidney Disease: Improving Global Outcomes; PICOS: Population, Intervention, Comparator, Outcomes, Study Design

Domain	Inclusion	Exclusion
Population	Hospitalised adults or children (general wards or ICU) without AKI at prediction time; unselected by a single diagnosis, procedure or drug exposure	Populations restricted to one disease (e.g., sepsis, heart failure, trauma), procedure (e.g., cardiac surgery, contrast) or drug exposure; outpatients
Intervention/Exposure	Multivariable AKI prediction or early warning model (statistical or machine learning), static or continuously updated	Single biomarkers; univariable scores; models without a fixed horizon
Comparator	Any (alternative models, clinical scores, usual care) or none	Not applicable
Outcomes	Incident AKI (any stage or stage 2–3, KDIGO or equivalent) at a fixed 24-h or 48-h horizon, with AUROC reported for that horizon	Other horizons only; horizon-specific AUROC not reported; AKI recovery, persistence, dialysis, or mortality only
Study design	Peer-reviewed primary studies (retrospective/prospective cohort, including case-control sampling within a cohort, external validation, implementation) published in English in 2021–2026	Reviews, meta-analyses, protocols, conference abstracts, editorials, letters, preprints; non-English publications

Information Sources and Search Strategy

PubMed, Web of Science, Scopus, Embase and Cumulative Index to Nursing and Allied Health Literature (CINAHL) were searched from January 1, 2021, to September 2026; the last search was run on September 19, 2026. The search combined terms for AKI, risk prediction, the 24-48-hour pre-onset window and machine learning or statistical modelling. Equivalent strategies using database-specific subject headings and field tags were applied in Web of Science, Scopus, Embase, and CINAHL; the full strategies for all five databases are given in the Appendices.

Study Selection

Records from all databases were imported into EndNote X6 (Clarivate, London, UK), and duplicates were removed. Two reviewers (SE & HAGF) independently screened the remaining records by title and abstract, and potentially relevant reports were retrieved and assessed in full text against the PICOS criteria, again independently by two reviewers. Disagreements were resolved by discussion or, when needed, by a third reviewer (MAOF). Reasons for exclusion at the full-text stage were recorded (Figure 1).

**Figure 1:**
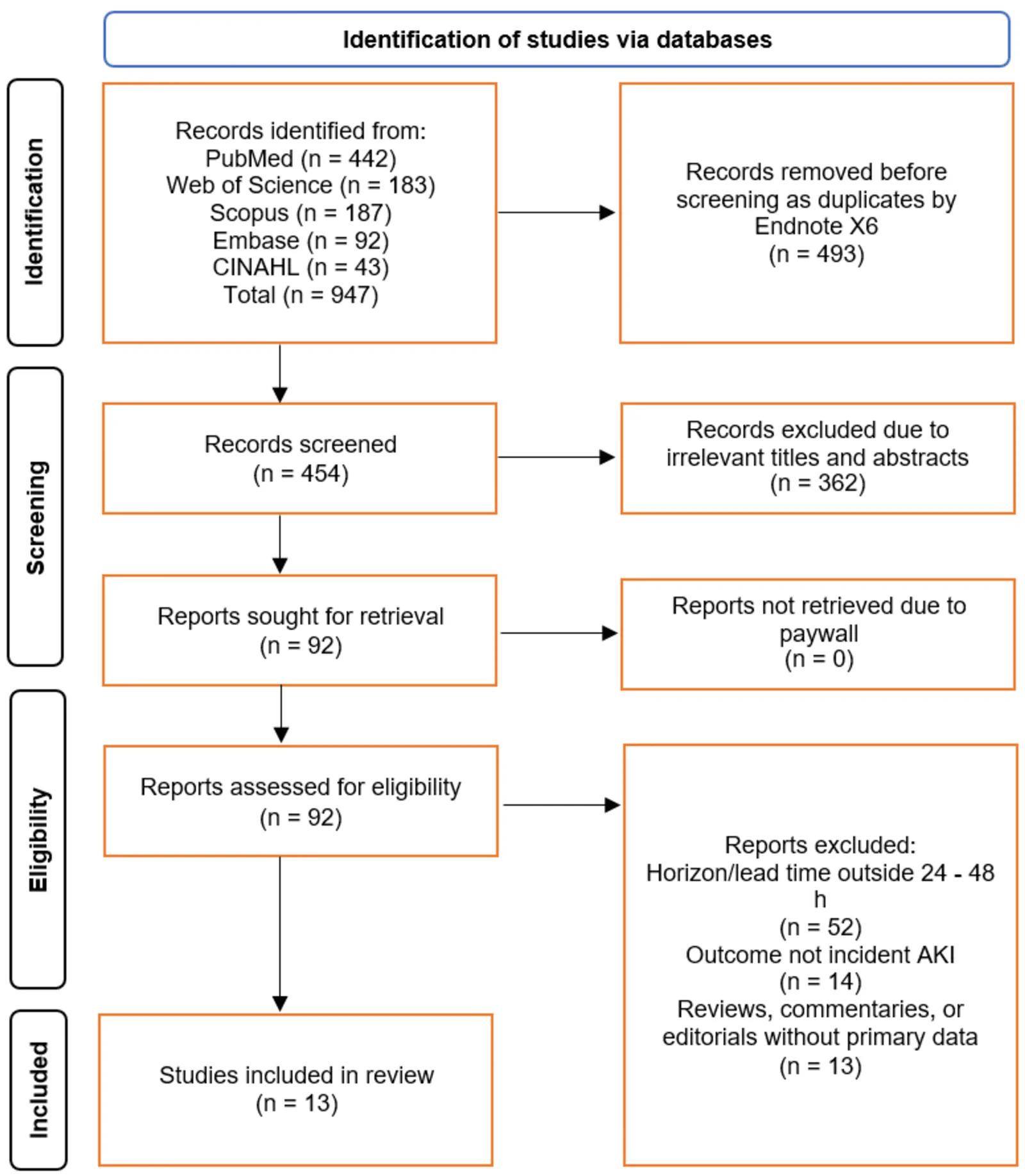
PRISMA 2020 flow diagram of study selection PRISMA: Preferred Reporting Items for Systematic Reviews and Meta-Analyses; CINAHL: Cumulative Index to Nursing and Allied Health Literature; AKI: acute kidney injury

Data Extraction

Data were extracted with a form based on the Checklist for Critical Appraisal and Data Extraction for Systematic Reviews of Prediction Modelling Studies (CHARMS) [11] by two reviewers working independently, with discrepancies resolved by consensus. Extracted items were first author, year, country or data source, design, setting and population, sample size, AKI definition, prediction horizon, update mode, algorithm, comparator, validation type, discrimination, calibration and implementation findings. When several models were reported, the best-performing model at the 24- or 48-hour horizon was extracted, because this is usually the model that authors carry forward for validation or use; as this rule may favour optimistic estimates, it is acknowledged as a limitation.

Risk of Bias Assessment

Risk of bias was assessed with the Prediction model Risk Of Bias ASsessment Tool (PROBAST) across four domains: participants, predictors, outcome and analysis [12], independently by two reviewers (SE & HAGF), with disagreements resolved by consensus. Adherence to TRIPOD+AI (Transparent Reporting of a multivariable prediction model for Individual Prognosis Or Diagnosis+artificial intelligence) reporting items informed judgements in the analysis domain [13]. The analysis domain was rated low when a model was evaluated in external, temporal, or prospective data with an adequate number of outcome events. It was rated unclear when only internal validation was performed, or key analysis details were missing, and high when data leakage or overfitting was likely. Overall risk of bias was rated high if any domain was high, low if all domains were low, and unclear otherwise.

Synthesis

Clinical and methodological heterogeneity was substantial: horizons, outcome stages, units of analysis, and validation strategies all differed. For this reason, results were synthesised narratively and in tables without statistical pooling. Because no estimates were pooled, heterogeneity statistics were not applicable, and the AUROC ranges reported below are descriptive rather than directly comparable estimates; 95% confidence intervals (CIs) are given where the source studies reported them. Discrimination estimates were displayed graphically.

Results

Study Selection

The database searches identified 947 records: 442 from PubMed, 183 from Web of Science, 187 from Scopus, 92 from Embase, and 43 from CINAHL (Figure 1). EndNote X6 removed 493 duplicates, leaving 454 records for title and abstract screening. Of these, 362 were excluded as irrelevant, and 92 reports were sought for retrieval; all were retrieved. After full-text assessment, 79 reports were excluded. Fifty-two had a prediction horizon or lead time outside 24-48 hours, 14 did not predict incident AKI, and 13 were reviews, commentaries, or editorials without primary data. Thirteen studies met all eligibility criteria and were included [14-26]. Yu et al. used case-control sampling within a hospital cohort and were retained, with this design reflected in its risk-of-bias rating [22]. Kim et al. was retained because its cohort was not restricted by diagnosis, procedure, or drug exposure; urine-output monitoring was a data-availability requirement rather than a clinical selection criterion, and the incomplete cohort description was reflected in its risk-of-bias rating [23].

Characteristics of Included Studies

Table 2 summarises the included studies. They were published between 2021 and 2026, and seven of the 13 appeared in 2025-2026. Data came from the United States [14,16,19,25], China [21,22,24], Taiwan [18], Singapore [15] and Sweden [20], and two studies combined data from several countries [17,26]. Six studies were set in ICUs [14,17,18,20,25,26] and six in general or hospital-wide inpatient populations [15,16,19,21,22,24]; one of the latter involved children [24]. One study did not report its setting [23]. Across the 12 studies that reported sample size, about 623,000 admissions or encounters were analysed; units of analysis differed between studies. All studies used retrospective EHR data for model development, and Liang et al. added a prospective validation cohort [17].

**Table 2:** Characteristics of included studies (n = 13) AKI: acute kidney injury; Retro: retrospective; prosp.: prospective; ext. val.: external validation; enc.: encounters; adm.: admissions; hosp.: hospitalisations; pts: patients; NR: not reported; CNN: convolutional neural network; RNN: recurrent neural network; LR: logistic regression; GBDT: gradient boosting decision tree; RF: random forest; NN: neural network; SVM: support vector machine; BiGRU: bidirectional gated recurrent unit; HA-AKI: hospital-acquired AKI; UO: urine output; MGB: Mass General Brigham; PLA GH: Chinese PLA General Hospital; NL: Netherlands

Study	Country/data	Design	Population	N	Model (exposure)
Le 2021 [14]	USA (MIMIC-III)	Retro	Adult ICU	12,347 enc.	CNN + clinical text
Chua 2021 [15]	Singapore	Retro	Adult inpatients	20,732 adm.	Bidirectional RNN
Liu 2022 [16]	USA; 1 centre	Retro	Adult inpatients	76,957 enc.	Personalised LR + transfer learning
Liang 2022 [17]	China, USA, NL	Retro + prosp.	Adult ICU	58,492 pts	GBDT (dynamic vitals/labs)
Huang 2023 [18]	Taiwan; 5 hospitals	Retro, federated	Adult ICU	16,732 + 38,424 adm.	XGBoost / NN / RF
Dutta 2024 [19]	USA (MGB)	Retro ext. val.	Adult inpatients	39,891 stays	Epic HA-AKI model
Koozi 2025 [20]	Sweden; 4 ICUs	Retro	Adult ICU	4,732 adm.	XGBoost (explainable AI)
Zhang Y 2025 [21]	China; multicentre	Retro	Adult inpatients	161,876 adm.	Random forest, 20 variables
Yu 2025 [22]	China (PLA GH)	Retro, case-control	Adult inpatients	59,936 + 4,022	XGBoost / SVM
Kim 2025 [23]	NR	Retro	Patients with UO monitoring	NR	Multi-task deep learning
Zhang C 2025 [24]	China; 2 children's hospitals	Retro	Paediatric inpatients	36,828 hosp.	Stacked XGBoost/LightGBM/CatBoost
Hourani 2026 [25]	USA (MIMIC-IV, eICU)	Retro	Adult ICU	51,833 + 6,339	XGBoost
Tang 2026 [26]	China, USA	Retro	Adult ICU	28,099 + 5,916	BiGRU + CNN + attention

Prediction Design and Algorithms

Model design and performance are shown in Table 3. Nine studies used a 48-hour horizon [14,15,17,19-23,26] and four used a 24-hour horizon [16,18,24,25]. At least six models were updated repeatedly during admission, from every six hours to every 12 hours or in real time [19,21-24,26], whereas Koozi et al. made a single prediction at ICU admission [20]. Gradient-boosted trees were the most common algorithm [18,20,22,25], followed by recurrent, convolutional and attention-based neural networks [14,15,23,26]. Other approaches included random forest [21], stacked ensembles [24], personalised logistic regression with transfer learning [16], and a proprietary commercial model [19].

**Table 3:** Prediction design and model performance AUROC values are for the 24- or 48-hour horizon of the best reported model (95% CI where reported by the source study). Huang et al.: the internal value is for the parsimonious model that was externally validated. The Brier score is an overall accuracy measure and was not counted as calibration. –: not applicable; NR: not reported; AUROC: area under the receiver operating characteristic curve

Study	Update mode	Validation	AUROC internal	AUROC external	Calibration
Le 2021 [14]	Pre-onset window	Internal	0.856	–	NR
Chua 2021 [15]	Longitudinal	Internal	0.81 (0.78–0.85)	–	NR
Liu 2022 [16]	Single point	Internal	0.78	–	NR
Liang 2022 [17]	Dynamic	Ext. + prospective	0.86	0.86; 0.84 (prosp.)	NR
Huang 2023 [18]	NR	External (4 sites)	0.911	0.760–0.865	Plot; risk overestimated
Dutta 2024 [19]	Repeated in-stay	External	–	0.76 (encounter 0.77)	NR
Koozi 2025 [20]	Admission (static)	Internal	0.76	–	NR
Zhang Y 2025 [21]	Real-time	Internal + 5 ext.	0.85 (0.84–0.86)	0.74–0.81	Good (all sites)
Yu 2025 [22]	12-h windows	Internal + ext.	0.936	0.823–0.833	NR (Brier ~0.2)
Kim 2025 [23]	6-hourly	Internal	0.993	–	NR
Zhang C 2025 [24]	Real-time	Internal + ext.	0.851	0.869	NR
Hourani 2026 [25]	NR	Temporal + geographic	0.88 (0.84–0.90)	0.84; 0.82	NR
Tang 2026 [26]	6-hourly	External (2)	0.720	0.577; 0.798	NR

Discrimination and Calibration

AUROCs are compared in Figure 2. In development or internal validation, AUROCs ranged from 0.72 [26] to 0.993 [23]. Kim et al. reported 0.993 without external validation, and such a value warrants caution [23]. In external, temporal, or prospective validation, AUROCs ranged from 0.577 to 0.869. External estimates were equal to or lower than internal estimates in every study that reported both [17,18,21,22,25,26], except for a small paediatric external cohort [24]. Tang et al. showed the largest drop between sites: 0.720 internally, 0.577 in eICU and 0.798 in a Chinese hospital [26]. In the only independent evaluation of a commercial vendor model, the Epic hospital-acquired AKI model achieved an AUROC of 0.76 for 48-hour prediction, below the vendor's own estimates [19]. Two studies reported calibration: Zhang et al. found good calibration at all sites [21], and Yu et al. reported Brier scores of about 0.2 [22].

**Figure 2:**
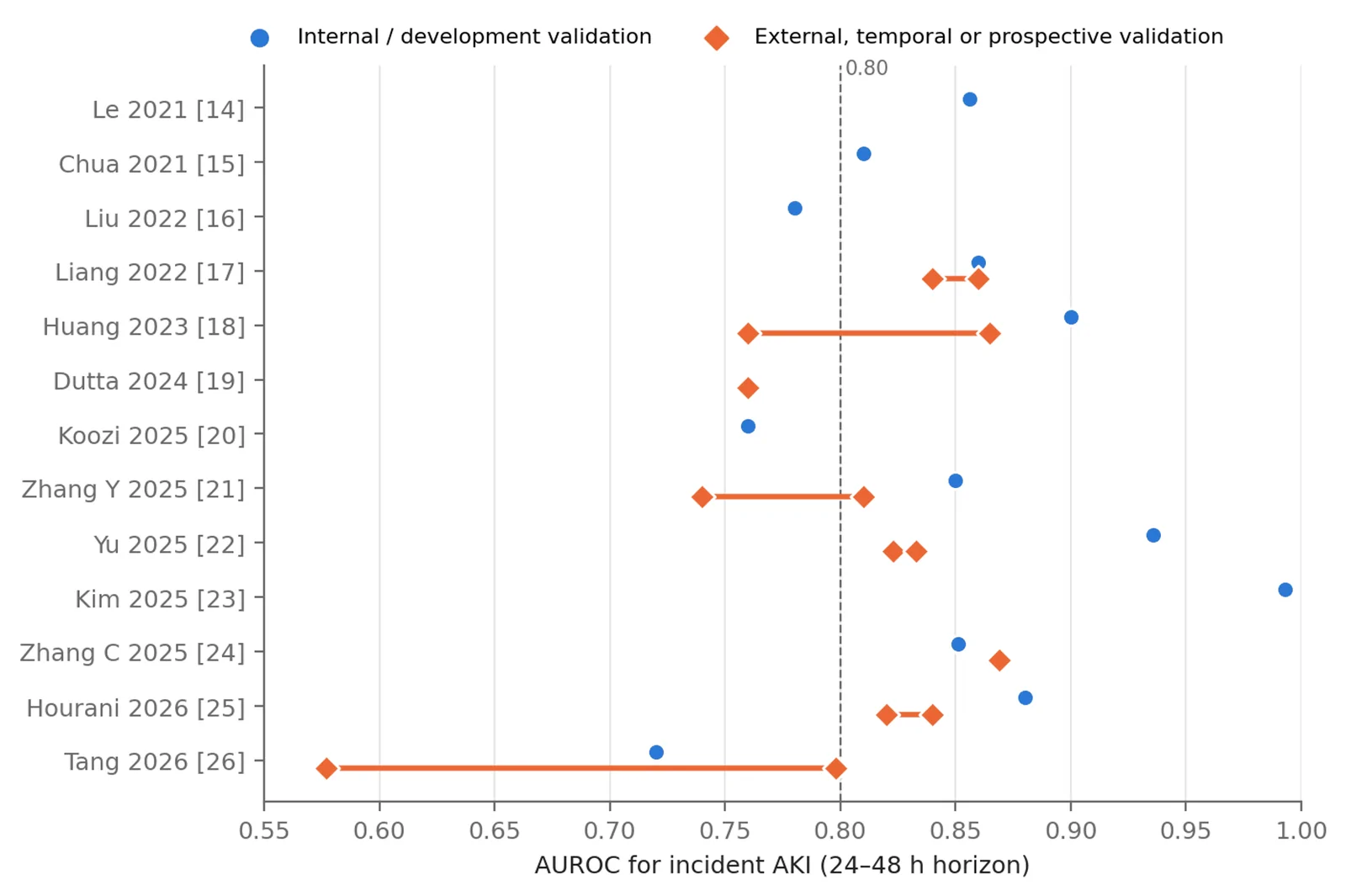
AUROC of AKI early warning models at the 24-48-hour horizon Circles: internal/development validation; diamonds: external, temporal, or prospective validation; lines join the range across external cohorts. The dashed vertical line marks an AUROC of 0.80, a conventional threshold for good discrimination, shown for visual reference only. Numbers in brackets are reference numbers. AUROC: area under the receiver operating characteristic curve; AKI: acute kidney injury [14-26]

Calibration

Only two studies formally assessed calibration. Zhang et al. found good calibration at all sites [21], whereas the calibration plots of Huang et al. showed that risk was overestimated [18]. Yu et al. reported Brier scores of about 0.2 [22]; because the Brier score combines discrimination and calibration, it was not counted as a calibration assessment.

Clinical Utility

Few studies measured clinical usefulness directly. Zhang et al. reported median lead times of 54-90 hours at external sites and a negative predictive value of 99.6%, but a positive predictive value of only 24.1% [21]. The Epic model warned a median of 21.6 hours before stage 1 AKI and was better at ruling out low-risk patients than at identifying high-risk ones [19]. Liu et al. showed that personalised models improved AUROC by up to 0.13 in high-risk subgroups [16]. No study reported a randomised or controlled evaluation of patient outcomes.

Risk of Bias

PROBAST judgements are shown in Figure 3. Overall risk of bias was low in five studies [17,19,21,25,26], unclear in six [14,15,16,18,20,24] and high in two [22,23]. Most unclear ratings came from the analysis domain, because models were validated only internally, or from the outcome domain, because the AKI definition was incompletely reported [15,18,24]. Yu et al. was rated high risk because of 1:1 case-control sampling in the development cohort [22]. Kim et al. was rated high risk because the cohort was not described and the near-perfect AUROC suggests possible overfitting [23]. The predictor domain was rated low in all studies except Kim et al. [23].

**Figure 3:**
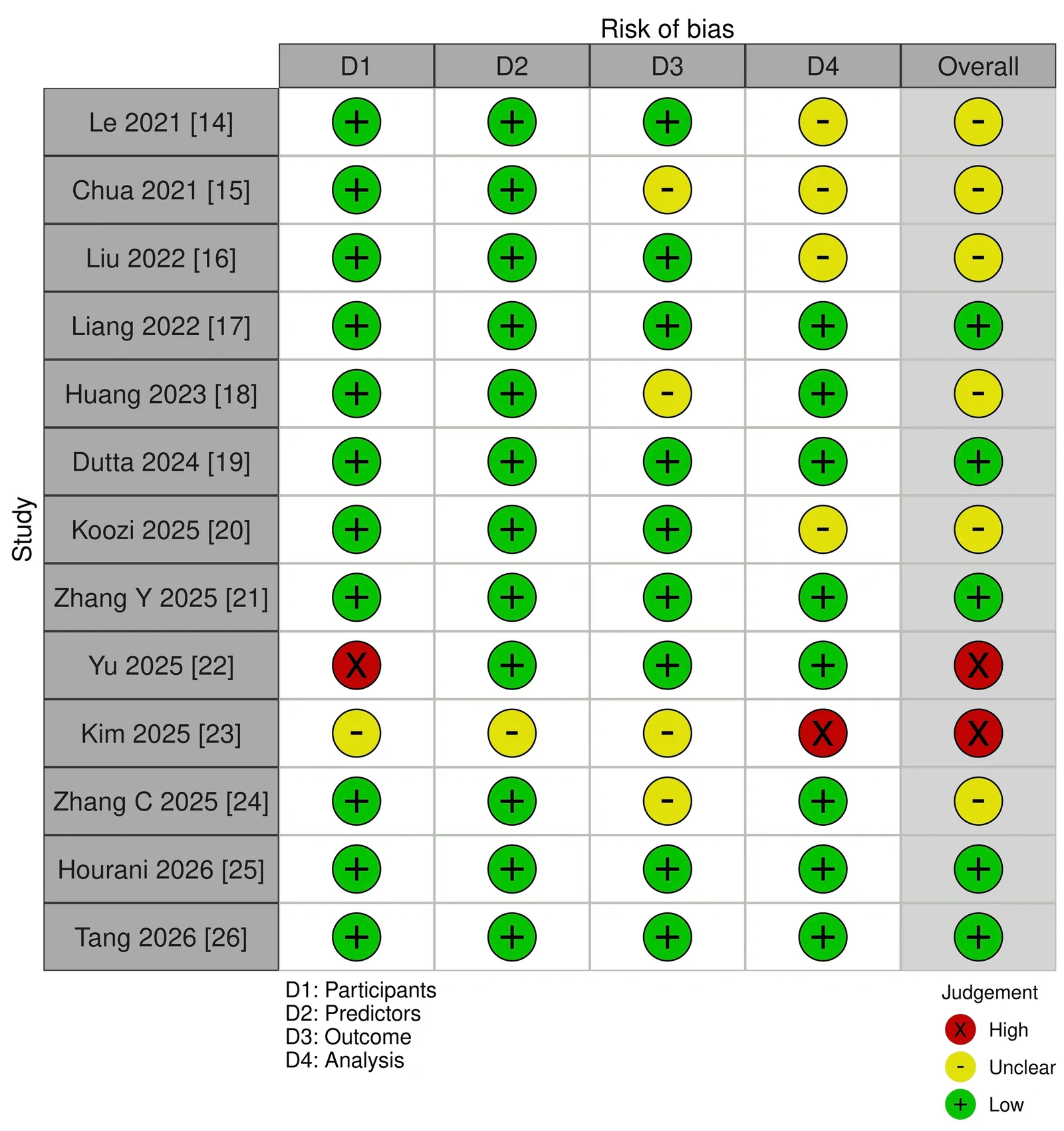
PROBAST risk of bias assessment PROBAST: Prediction model Risk Of Bias ASsessment Tool [14-26]

Discussion

This systematic review identified 13 original studies, published between 2021 and 2026, of models that predict AKI 24-48 hours before it meets diagnostic criteria. The main finding is consistent: models achieve moderate-to-excellent discrimination in the data they were built on, but performance falls when they are moved to new hospitals, periods, or populations. Internal AUROCs mostly lay between 0.76 and 0.94, while external AUROCs lay between 0.58 and 0.87, and the external estimate was equal to or lower in five of the seven studies that reported both. The evidence also shows a gap between prediction and prevention. Only one study included prospective validation [17], only one independently evaluated a model already running in routine care [19], and none tested whether acting on an alert changes patient outcomes. This absence of outcome evidence is the most clinically important finding of this review. AKI prediction models have therefore matured technically, but their value as clinical early warning systems remains largely unproven.

These findings build on the models that shaped the field before 2021. Koyner et al. developed a gradient-boosted inpatient AKI model that predicted stage 2 AKI within 48 hours with an AUROC of about 0.87 [7]. Tomašev et al. showed that a recurrent neural network could provide continuous 48-hour predictions for United States veterans, although that population was 94% male [8]. Several included studies continue these lines of work. Zhang et al. showed that a 20-variable random forest transported moderately well to five Chinese hospitals and improved further when refitted locally [21]. Hourani et al. showed that a gradient-boosted ICU model kept an AUROC above 0.80 in both temporal and geographic validation [25]. Huang et al. showed that federated learning allowed Taiwanese hospitals to train a shared model without pooling patient data while keeping external AUROCs between 0.76 and 0.87 [18]. Together, these studies suggest that parsimonious models and local recalibration matter more for transportability than architectural complexity.

Our results are consistent with earlier systematic reviews. Vagliano et al. appraised machine learning models for AKI and found that most were at high risk of bias, rarely externally validated, and seldom reported calibration [27]. Wainstein et al. reviewed only externally validated models in general hospital populations and also found a drop in discrimination on external testing and limited evidence of clinical benefit [28]. The present review adds three things. First, it focuses on the fixed 24-48-hour horizon that matters clinically, instead of combining models with lead times from minutes to weeks. Second, it includes studies published through 2026, including federated, stacked-ensemble, and attention-based approaches. Third, it includes a real-world evaluation of a commercial model. Five of the 13 studies were at low risk of bias, suggesting that methodological rigour in this field is improving, especially through the use of external and temporal validation.

The drop in external performance is expected and has several causes. AKI definitions differed between studies: most used KDIGO creatinine criteria, some added urine output, and several reported the definition incompletely [15,18,24]. Because urine output is recorded reliably mainly in the ICU, ward-based and ICU-based models are not interchangeable. Baseline creatinine was estimated differently, which changes who is classified as having AKI and when. Case mix, testing frequency, and documentation also vary between hospitals and over time, and models trained on frequent ICU measurements may perform worse where laboratory tests are sparser. The large between-site variation reported by Tang et al. [26] and the lower AUROC of the Epic model at Mass General Brigham compared with the vendor's own estimates [19] illustrate this. Taken together, these examples argue strongly for local validation before any model is switched on.

Calibration was the weakest part of the evidence. Only two studies formally assessed calibration, even though calibration matters as much as discrimination when a probability triggers an alert. A model that ranks patients well but overestimates risk, as observed by Huang et al. [18], will produce too many alerts and contribute to alert fatigue, and one that underestimates risk gives false reassurance. Calibration has been called the Achilles heel of predictive analytics for this reason [29]. Zhang et al. showed that calibration can be preserved across several sites [21], which is encouraging, but calibration in important subgroups, such as women, older adults and patients with chronic kidney disease, was not examined in any included study. Future studies should report calibration slopes, intercepts and plots at each validation site and for important subgroups, as TRIPOD+AI recommends [13].

Horizon choice involves a trade-off. A 24-hour horizon gives some time for simple actions such as stopping a nephrotoxin or reviewing fluid balance. A 48-hour horizon gives more lead time but usually lower accuracy, because the signal of impending injury is weaker further from onset. Zhang et al. reported both horizons and showed that 48-hour predictions still gave median lead times of 54-90 hours at external sites [21], while the Epic model warned about 22 hours before stage 1 AKI despite its nominal 48-hour horizon [19]. The best horizon probably depends on what clinicians can do in response. A nephrotoxin stewardship prompt may need only 24 hours, whereas preventive haemodynamic optimisation in the ICU may benefit from longer notice. Few included studies linked their horizon to a specific clinical action, and future work should do so explicitly.

Positive predictive value limits usefulness in practice. AKI is relatively uncommon at any single prediction time, so even a model with an AUROC above 0.85 will produce many false positives. Zhang et al. reported a negative predictive value of 99.6%, but a positive predictive value of 24.1% at the optimal threshold [21], and the Epic model was better at ruling out risk than at identifying high-risk patients [19]. Le et al. similarly reported a positive predictive value of about 0.24 for their 48-hour convolutional model [14]. This matters because randomised evidence shows that alerting clinicians to established AKI does not improve outcomes by itself [9]. Early warnings should therefore be paired with specific, low-harm actions, such as nephrotoxin review or pharmacist-led dose adjustment, and be judged by those actions and patient outcomes rather than by AUROC alone.

Interpretability, personalisation and privacy also emerged as themes. Koozi et al. used explainable AI methods, and Tang et al. used Shapley additive explanations to show which features drove predictions [20,26], and Yu et al. identified uric acid, colloid administration, admission creatinine, pulse and albumin as leading predictors [22]. Liu et al. used transfer learning to personalise risk estimates and improved AUROC by up to 0.13 in high-risk subgroups such as liver transplant and cardiac surgery patients [16]. Chua et al. modelled both time-invariant and time-varying features in a bidirectional recurrent network, allowing risk to be tracked as admission unfolds [15]. Huang et al. showed that federated learning could protect privacy across hospitals [18]. These approaches may improve clinical trust, but none has yet been shown to change clinician behaviour or patient outcomes.

Some populations are under-represented. Only one included study involved children [24]. Paediatric AKI differs from adult AKI in baseline creatinine estimation, aetiology and treatment responses, so adult models cannot be assumed to transfer. The paediatric model of Zhang et al. performed well, but its external cohort was small (462 patients) [24]. Low- and middle-income countries were not represented, even though much of the global AKI burden occurs there, often outside ICUs and with limited laboratory monitoring [1,3]. Models that depend on frequent laboratory tests are unlikely to work in such settings without adaptation, and simpler models built on routinely available variables may be more suitable.

The comparators used in the included studies also say something about the value these models add. Most studies compared one algorithm with another, and Le et al. compared their convolutional model with the Sequential Organ Failure Assessment score, which it clearly outperformed [14]. Head-to-head comparison with experienced clinicians was absent. Algorithm-versus-algorithm comparisons show technical progress but not whether a model adds to what clinicians already see at the bedside. Future evaluations should use clinically meaningful comparators, such as existing alert rules, nephrology review, or structured clinical assessment, and should report net benefit across realistic alert thresholds. Only then can hospitals judge whether the extra lead time justifies the workload an early warning system creates.

Beyond the absence of trial evidence, several knowledge gaps remain: standard definitions of prediction time, horizon, and AKI onset are needed so that models can be compared; calibration, subgroup performance, and decision-curve analysis should be reported routinely; deployed models should be monitored for drift; and combining EHR-based risk scores with kidney stress biomarkers should be tested as a way to improve positive predictive value, particularly in the ICU.

For clinicians and health systems, the practical message is cautious optimism. Tools that predict AKI 24-48 hours ahead are technically feasible, and several perform well across sites. However, a health system considering one, whether developed in-house or bought from a vendor, should first validate it locally, check calibration and subgroup performance, choose an alert threshold that its staff can respond to, and link each alert to a defined preventive action. Staged implementation with continued outcome monitoring, ideally in a pragmatic trial, is the most defensible route from prediction to patient benefit.

Limitations

This review has limitations. The protocol was not prospectively registered, which increases the risk of undetected changes to the methods. Although all 92 full-text reports were retrieved, supplementary material was not accessible for one study, so its calibration and secondary performance measures may be under-reported [23]. Extracting the best-performing model from each study may have favoured optimistic discrimination estimates. Heterogeneous horizons, AKI definitions and units of analysis precluded meta-analysis. Restricting the review to 2021-2026 excluded influential earlier models, and excluding disease- or procedure-specific models limits generalisation to those settings. Grey literature, preprints, and non-English publications were excluded, so publication and language bias cannot be excluded.

## Conclusions

Models that predict AKI 24-48 hours before clinical onset show moderate-to-good discrimination in unselected hospitalised and ICU populations, but performance falls off on external validation. Calibration is rarely reported, and there is no trial evidence of improved patient outcomes. Before routine use, AKI early warning systems need local validation, transparent calibration and subgroup reporting, and prospective evaluation of whether their alerts lead to effective preventive action.

## Appendices

**Table 4:** Search strategy

Database	Search String
PubMed/MEDLINE	( "Acute Kidney Injury"[Mesh] OR "Acute Kidney Failure"[Mesh] OR "acute kidney injur*"[tiab] OR "acute renal injur*"[tiab] OR "acute renal failure"[tiab] OR AKI[tiab] ) AND ( "Risk Assessment"[Mesh] OR "Risk Prediction"[tiab] OR "risk stratification"[tiab] OR "risk prediction"[tiab] OR "risk assessment"[tiab] OR "prediction model*"[tiab] OR "predictive model*"[tiab] OR "prediction algorithm*"[tiab] OR "predictive algorithm*"[tiab] OR "prognostic model*"[tiab] OR "clinical prediction model*"[tiab] OR "risk score*"[tiab] OR "prediction score*"[tiab] OR "early warning"[tiab] OR "early warning system*"[tiab] OR "early detection"[tiab] OR "early identification"[tiab] OR "early prediction"[tiab] OR "forecast*"[tiab] OR "predict*"[tiab] ) AND ( "before onset"[tiab] OR "prior to onset"[tiab] OR "pre-onset"[tiab] OR "before development"[tiab] OR "prior to development"[tiab] OR "preclinical"[tiab] OR "24 hour*"[tiab] OR "24-hour*"[tiab] OR "48 hour*"[tiab] OR "48-hour*"[tiab] OR "24 to 48 hour*"[tiab] OR "24-48 hour*"[tiab] OR "24 h"[tiab] OR "48 h"[tiab] ) AND ( "Machine Learning"[Mesh] OR "Artificial Intelligence"[Mesh] OR "machine learning"[tiab] OR "deep learning"[tiab] OR "artificial intelligence"[tiab] OR "neural network*"[tiab] OR "random forest*"[tiab] OR "decision tree*"[tiab] OR "gradient boosting"[tiab] OR "XGBoost"[tiab] OR "logistic regression"[tiab] )
Embase (Embase.com)	#1 'acute kidney failure'/exp OR ('acute kidney injur*' OR 'acute renal injur*' OR 'acute renal failure' OR aki):ti,ab; #2 'risk assessment'/exp OR 'prediction'/exp OR ('risk prediction' OR 'risk stratification' OR 'risk assessment' OR 'prediction model*' OR 'predictive model*' OR 'prediction algorithm*' OR 'predictive algorithm*' OR 'prognostic model*' OR 'clinical prediction model*' OR 'risk score*' OR 'prediction score*' OR 'early warning' OR 'early warning system*' OR 'early detection' OR 'early identification' OR 'early prediction' OR forecast* OR predict*):ti,ab; #3 ('before onset' OR 'prior to onset' OR 'pre-onset' OR 'before development' OR 'prior to development' OR preclinical OR '24 hour*' OR '24-hour*' OR '48 hour*' OR '48-hour*' OR '24 to 48 hour*' OR '24-48 hour*' OR '24 h' OR '48 h'):ti,ab; #4 'machine learning'/exp OR 'artificial intelligence'/exp OR ('machine learning' OR 'deep learning' OR 'artificial intelligence' OR 'neural network*' OR 'random forest*' OR 'decision tree*' OR 'gradient boosting' OR xgboost OR 'logistic regression'):ti,ab; #5 #1 AND #2 AND #3 AND #4 AND (2021-2026)/py.
Scopus	TITLE-ABS-KEY("acute kidney injur*" OR "acute renal injur*" OR "acute renal failure" OR "acute kidney failure" OR AKI) AND TITLE-ABS-KEY("risk prediction" OR "risk stratification" OR "risk assessment" OR "prediction model*" OR "predictive model*" OR "prediction algorithm*" OR "predictive algorithm*" OR "prognostic model*" OR "risk score*" OR "prediction score*" OR "early warning*" OR "early detection" OR "early identification" OR "early prediction" OR forecast* OR predict*) AND TITLE-ABS-KEY("before onset" OR "prior to onset" OR "pre-onset" OR "before development" OR "prior to development" OR preclinical OR "24 hour*" OR "24-hour*" OR "48 hour*" OR "48-hour*" OR "24-48 hour*" OR "24 h" OR "48 h") AND TITLE-ABS-KEY("machine learning" OR "deep learning" OR "artificial intelligence" OR "neural network*" OR "random forest*" OR "decision tree*" OR "gradient boosting" OR XGBoost OR "logistic regression") AND PUBYEAR > 2020.
Web of Science Core Collection	TS=("acute kidney injur*" OR "acute renal injur*" OR "acute renal failure" OR "acute kidney failure" OR AKI) AND TS=(the same risk-prediction terms as Scopus) AND TS=(the same pre-onset/time-window terms as Scopus) AND TS=(the same machine learning/statistical modelling terms as Scopus); publication years 2021–2026.
CINAHL (EBSCOhost)	(MH "Acute Kidney Injury+" OR TI/AB "acute kidney injur*" OR "acute renal injur*" OR "acute renal failure" OR AKI) AND (MH "Risk Assessment" OR TI/AB risk-prediction terms as above) AND (TI/AB pre-onset/time-window terms as above) AND (MH "Machine Learning" OR MH "Artificial Intelligence+" OR TI/AB machine learning terms as above); published 2021–2026.
